# Sexual risk after HIV diagnosis: a comparison of pre-ART individuals with CD4>500 cells/µl and ART-eligible individuals in a HIV treatment and care programme in rural KwaZulu-Natal, South Africa

**DOI:** 10.7448/IAS.16.1.18048

**Published:** 2013-08-05

**Authors:** Nuala McGrath, Linda Richter, Marie-Louise Newell

**Affiliations:** 1 Academic Unit of Primary Care and Population Sciences, and Division of Social Statistics and Demography, University of Southampton, Southampton, UK; 2 Africa Centre for Health and Population Studies, University of KwaZulu-Natal, Somkhele, South Africa; 3 HIV, STIs and TB programme, Human Sciences Research Council, Durban, South Africa; 4 Developmental Pathways for Health research unit, University of the Witwatersrand, Johannesburg, South Africa; 5 School of Applied Human Sciences, University of KwaZulu-Natal, Durban, South Africa; 6 Faculty of Medicine, University of Southampton, Southampton, UK

**Keywords:** AIDS, antiretroviral therapy, HIV, sexual risk behaviours, ART-eligible, pre-ART

## Abstract

**Introduction:**

Little is known about people diagnosed as HIV-positive who access HIV care early in their disease. In pre-ART studies published to date, only a minority of the participants have CD4>500 cells/µl.

**Methods:**

This cross-sectional study compared individuals presenting for HIV care with CD4>500 cells/µl, “pre-ART” (*N*=247), with individuals who had CD4<200 cells/µl or WHO Stage IV, “ART-eligible” (*N*=385). Baseline characteristics were contrasted between the two groups and logistic regression models used to explore group differences in: (a) being sexually active in the last month; (b) disclosure of HIV status to current partner; (c) knowing the HIV status of one's current partner; and (d) condom use at last sex.

**Results:**

Pre-ART and ART-eligible individuals were similar in terms of a wide range of socio-demographic characteristics. Controlling for gender, only current sexual behaviour and HIV-testing history were significantly different between ART groups. In multivariable models, participants in the pre-ART group were twice as likely to be sexually active in the last month, OR 2.06 95% CI (1.32, 3.21), and to know their partner's status, OR 1.95 (1.18, 3.22) compared to those in the ART-eligible group. Self-reported disclosure of HIV status to current sexual partner (71%), condom use at last sex (61%) and HIV concordancy within relationships were not significantly different between the two ART groups. Overall, 39% of the study participants reported knowing that they were in concordant HIV-positive relationships. Fifty-five percent of all participants reported not knowing their partner's HIV status, only half of whom reported using a condom at last sex. Pre-ART individuals were significantly less likely to have tested HIV-positive for the first time in the last year and to have tested for sickness-related reasons than the ART-eligible group.

**Conclusions:**

Reported sexual risk behaviours by pre-ART individuals with CD4>500 cells/µl suggest a continued risk of onward HIV transmission. There is a need for positive prevention efforts to target this group given that current treatment guidelines do not provide them with ART. Strengthening support regarding disclosure, partner HIV testing and consistent condom use, and further promotion of HIV testing in the community to assist earlier diagnosis are urgently required.

## Introduction

People diagnosed as HIV-positive who have accessed HIV care but are not yet eligible for antiretroviral treatment (pre-ART) have received little attention other than as a comparison group for assessing the impact of antiretroviral treatment (ART) on sexual behaviour [1–4], economic activities [5] or more recently their attrition from HIV care clinics [6–9]. HIV-positive individuals are more likely to be partnered, be sexually active, have unprotected sex and more than one sex partner during the pre-ART period than post-ART initiation [1,10]. However, in studies published to date pre-ART is usually defined by CD4>200 cells/µl and only a minority have CD4>500 cells/µl.

Recently, recommendations for ART eligibility have expanded to include all HIV-infected individuals with CD4<350 cells/µl [11], and treatment with ART has been shown to substantially lower the probability of onward HIV transmission [12,13]. HIV-infected individuals with CD4>500 cells/µl thus remain potential contributors to transmission of HIV in the community. Although they may be less infectious at this earlier stage of disease and healthier than individuals with more advanced HIV disease, they do not benefit from viral load suppressive-ART. They are less likely to present at clinics for HIV care and less likely to attend regularly than pre-ART individuals with lower CD4 counts [6]. Individuals who access HIV care while having CD4>500 cells/µl can be considered to be a special group; insight into their characteristics may provide an opportunity for tailoring HIV prevention strategies.

A prospective cohort of HIV-infected individuals in the Hlabisa sub-district of uMkhanyakude in northern KwaZulu-Natal, South Africa, which aimed to investigate the impact of ART on family and partner relationships, and sexual behaviour, allows us to compare pre-ART individuals accessing the local HIV care programme with individuals newly identified as ART-eligible. This article aims to describe the characteristics of a pre-ART group of individuals who have CD4>500 cells/µl, and examine whether their socio-demographic and behavioural characteristics, their partnerships and current sexual behaviour differ from those of ART-eligible individuals.

## Methods

The Hlabisa HIV Treatment and Care Programme is a partnership between the Department of Health (in 17 Primary Health Care clinics, PHCs) and the Africa Centre for Health and Population Studies [14]. People first access care from the programme for HIV counselling and testing. Blood is taken for CD4 measurement after a positive HIV diagnosis, and, with clinical staging, used to determine ART eligibility. During follow-up clinic visits, pre-ART individuals receive individual counselling, with advice on healthy living, disclosure, partner notification and testing, transmission risk reduction measures and family planning. They are advised to return six months later for repeated clinical and CD4 count assessment. Since the inception of the programme in 2004 more than 50,000 HIV-positive individuals have accessed HIV services at least once, and approximately 25,000 initiated ART (Colin Newell, personal communication, June 2013). In part of the programme catchment area, the Africa Centre has collected longitudinal social, demographic and health data among a population of 90,000 since 2000 (see www.africacentre.ac.za) [15].

This article uses baseline data from a cohort study described in detail elsewhere [16]. In brief, men and women diagnosed as HIV-positive, accessing the HIV treatment and care programme in 3 of the 17 PHCs, aged 18 years or older and resident within the Africa Centre Demographic Surveillance Area (ACDSA), were eligible for this study. From January 2009, individuals were screened for study eligibility when they returned to the clinic to receive their CD4 test result. The criteria for the study's ART-eligible group was consistent with the national guidelines for ART-eligibility in 2009, a CD4<200 cells/µl or WHO Stage IV HIV disease. Individuals eligible for the study's pre-ART group had CD4>500 cells/µl; this cut-off was chosen to allow repeated measures of this group over time before they initiated treatment [16].

Individuals were enrolled after giving written informed consent, which included separate consent to link their study data to routine clinical data in the ART programme and the AC surveillance database (ACDIS). Once enrolled, a baseline questionnaire, focused on socio-demographic, behavioural and partnership-level characteristics, was administered in a private room by study staff not involved in the programme. The sexual risk behaviour information collected included details of up to three sexual partners in the last six months, sexual activity and condom use, and knowledge of partner's HIV status. The questionnaire also included adapted versions of a gender norms (*social expectations for appropriate behaviours of men as compared to women*) scale [17] and internalized HIV stigma scale [18], four statements measuring perceived self-efficacy (*people's beliefs about their capabilities to produce effects* [19]) and questions examining the level of social support and social capital available to individuals that derived from Myer *et al*. [20], and Pronyk *et al*. [21]. Matching participants to the ART programme database provided a history of clinic attendance for both groups and more detailed clinical information about TB diagnosis and laboratory tests. Matching participants to the ACDIS database provided migration histories [22].

The ethics committees of the University of KwaZulu-Natal (ref BF083/08), the London School of Hygiene and Tropical Medicine (ref. 5413), and the provincial Department of Health in Pietermaritzburg approved this study.

### Analysis

Baseline characteristics were compared between the two groups using Chi-square tests for categorical variables and Wilcoxon rank sum tests for continuous variables. Previous studies in the local area have shown that approximately 32% of ART-initiators are men, and that among pre-ART individuals, the proportion of males is even lower [6,23]. Thus, when statistically significant differences were identified between the two ART groups, the comparison was repeated within gender-specific groups to remove any confounding by gender.

Reasons for testing at the time of the participant's first HIV-positive test were grouped into self-initiated or not, and the self-initiated group was further divided into sickness- and non-sickness-related reasons. Gender norms items and internalized HIV stigma items had three options for each answer: agree, no opinion and disagree. The answers were coded and summed within each scale for a gender norm score (possible range: 19–57, higher scores indicating more support for equitable norms) and a stigma score (possible range is 24–72, a high score representing greater stigma) for each individual.

A priori, the exploration of group differences was of particular interest for four proximate determinants of onward transmission of HIV: (i) being sexually active in the month before baseline among those with at least one ongoing partnership at baseline; (ii) disclosing of HIV status to current partner by baseline; (iii) knowing the HIV status of one's current partner; and (iv) using condoms with a current partner among those who were sexually active in the last month. These outcomes were initially examined using logistic regression models for any association with study group, with and without adjustment for gender and age. For the subset of outcomes that were significantly different between study groups after adjusting for gender and age, group differences were explored in multivariable analysis. Age, gender and any baseline characteristics with a likelihood ratio test *p*-value of <0.05 were included in the final multivariable models. Continuous measures were considered for model inclusion as categorical variables represented by dummy indicators. Recruitment clinic, as a proxy for unmeasured characteristics of the participants that may have differed between clinic populations, was considered in each multivariable model. The models involved a participant-level analysis for outcome (i) and a partnership-level analysis for outcomes (ii)–(iv) with variance adjustment for correlation between participants’ multiple on-going partnerships. For partnership-level multivariable analyses, partner characteristics and relationship-level variables including fertility and fertility intentions, history of physical violence with current main partner, patterns of decision-making, and the length and quality of that relationship, were also considered as possible factors in the model. Wald tests were used to assess the contribution of each variable to the final partnership-level multivariable models. All statistical analyses were performed using STATA version 11.2 (StataCorp, College Station, TX, USA).

## Results

Recruitment ended March 2011, with 632 individuals enrolled: 247 in the pre-ART group (14% male) and 385 in the ART-eligible group (37% male). All but one participant was matched with the HIV treatment and care programme database and 505 (80%) were matched with ACDIS.

The two groups were similar in terms of age, marital status, education, parity, employment status, religion, socio-economic status, stigma scores, gender norms, and most aspects of social capital and support (Table 1). Crude differences in residential and employment histories, and receipt of a government grant were driven by gender differentials; males were more likely to have had a period of non-residence, been employed, and less likely to be receiving a government grant than women [24].

**Table 1:** Selected characteristics of study participants by ART group^a^

	Overall			Females			Males		
					
	ART-eligible (*N*=385)	Pre-ART (*N*=247)		ART-eligible (*N*=244)	Pre-ART (*N*=212)		ART-eligible (*N*=141)	Pre-ART (*N*=35)	
						
	*n* (% of *N*)	*n* (% of *N*)	*p* b	*n* (% of *N*)	*n* (% of *N*)	*p* c	*n* (% of *N*)	*n* (% of *N*)	*p* d
*Demographic*									
Age (years): median and interquartile range (IQR)	35 (29, 43)	34 (27, 43)	0.14						
Current marital status									
Never married	304 (79)	199 (81)	0.41						
Monog married	42 (11)	32 (13)							
Polygamously married	7 (2)	3 (1)							
Separated/divorced/widowed	32 (8)	13 (5)							
Religion									
None	50 (13)	19 (8)	0.26						
Shembe	71 (18)	48 (19)							
Zionist	120 (31)	74 (30)							
Western Christian	124 (32)	90 (36)							
Others	20 (5)	16 (6)							
Education: achieved matriculation or higher (Yes) (40 missing)	98 (28)	67 (28)	0.49						
Parity (females only): median (IQR) (18 missing)	2 (1, 4)	2 (2, 4)	0.85						
Number of children (men): median (IQR)	3 (1, 5)	3 (1, 4)	0.33						
Currently employed (Yes)	96 (25)	49 (20)	0.14						
Ever employed (Yes)	238 (62)	126 (51)	0.007	115 (47)	94 (44)	0.55	123 (87)	32 (91)	0.49
Participant receives at least one government grant (Yes)	207 (54)	180 (73)	<0.001	179 (73)	171 (81)	0.07	28 (20)	9 (26)	0.45
Length of time observed in surveillance (years)^e^	9.0 (9.0, 9.1)	9.0 (8.3, 10.0)	0.02	9.0 (8.9, 9.1)	9.0 (8.4, 10.0)	0.03	9.0 (9.0, 9.2)	9.2 (6.3, 9.7)	0.13
Past mobility, % of time spent as a non-resident:^e^ median% (IQR)	11 (0, 49)	0 (0, 31)	0.02	5.5 (0, 40)	0 (0,30)	0.17	23 (0, 67)	18.5 (0, 49.5)	0.44
Stigma score: median (IQR)	42 (36,48)	42 (36,48)	0.68						
Gender norms score: median (IQR)	37 (35, 41)	40 (37, 43)	0.16						
*Social capital*									
Neighbour contribute time for a project (Yes)	268 (70)	184 (75)	0.18						
Neighbour contribute money (R10) for a project (Yes)	259 (67)	173 (70)	0.47						
Who would deal with a problem that affected all of the neighbourhood?			<0.001			0.005			0.04
Municipal/district leaders	144 (37)	56 (23)		91 (37)	47 (22)		53 (38)	9 (26)	
Each person/household individually or neighbours	38 (10)	21 (9)		19 (8)	20 (10)		19 (13)	1 (3)	
Traditional leaders	116 (30)	89 (36)		74 (30)	77 (36)		42 (30)	12 (34)	
Traditional and municipal/district leaders would act together	87 (23)	81 (33)		60 (25)	68 (32)		27 (19)	13 (37)	
Are there community groups in your neighbourhood? (Yes)	337 (88)	187 (76)	<0.001	214 (88)	163 (77)	<0.002	123 (87)	24 (69)	0.008
How much can you rely on family/friends if you have a serious problem?			0.008			0.02			0.19
A little	77 (20)	27 (11)		52 (21)	24 (11)		25 (18)	3 (9)	
A lot	296 (77)	214 (87)		185 (76)	182 (86)		111 (79)	32 (91)	
Not at all	12 (3)	6 (2)		7 (3)	6 (3)		5 (4)	0 (-)	
How often do you spend time with neighbours?			0.001			0.01			0.03
Every day	97 (25)	83 (34)		62 (25)	71 (33)		35 (25)	12 (34)	
Several days a week	103 (27)	61 (25)		62 (25)	57 (27)		41 (29)	4 (11)	
At least once a fortnight	6 (2)	10 (4)		6 (2)	10 (5)		–	–	
Once a month	27 (7)	28 (11)		19 (8)	22 (10)		8 (6)	6 (17)	
Less than once a month/not at all	152 (39)	65 (26)		95 (39)	52 (25)		57 (40)	13 (37)	

a Gender-specific data are presented only when there is a significant difference between ART groups overall.

b,c,d A Chi-square test of ART-eligible vs. pre-ART; ^b^overall, ^c^among females, ^d^among males.

e Available for *N*=488. In the Africa Centre surveillance system, household membership is not conditional on residency, an individual can be recorded as a non-resident household member if they are residing in a household outside the demographic surveillance area (DSA) but remain socially connected to a household in the DSA. Changes in residence by individuals and households are documented within the DSA (internal migration) and into or out of the DSA (external migration) since January 2000.

Table 2 shows how HIV-testing history differs between groups. ART-eligible individuals were significantly more likely to have tested positive for the first time in the last year and for sickness-related reasons than the pre-ART group. These differences remained significant within gender and within the subset that tested HIV-positive on their first HIV test. Almost all men (93%) reported having self-initiated their HIV test, compared to 71% of women (*p*<0.001). In most instances where a woman did not self-initiate her test, the test took place in antenatal care. For ART-eligible individuals, the recruitment CD4 test was significantly more likely to represent the first interaction with the ART programme and a shorter length of time engaged in care than for pre-ART individuals. Despite these differences in testing history and previous interaction with the ART programme, the proportion who had disclosed their HIV status to anyone by baseline was not significantly different, 87%, *p*=0.58. Only 1% in each group reported being a member of an organization that supports people with HIV/AIDS (*p*=0.84), with no difference by recency of HIV diagnosis.

**Table 2:** Testing history by ART group

	Overall			Females			Males		
						
	ART-eligible (*N*=385)	Pre-ART (*N*=247)		ART-eligible (*N*=244)	Pre-ART (*N*=212)		ART-eligible (*N*=141)	Pre-ART (*N*=35)	
		
	*n* (% of *N*)	*n* (% of *N*)	*p* a	*n* (% of *N*)	*n* (% of N)	*p* b	*n* (% of *N*)	*n* (% of *N*)	*p* c
HIV-positive test was first ever HIV test (Yes)	328 (85)	182 (73)	<0.001	202 (83)	153 (72)	0.005	126 (89)	29 (83)	0.29
HIV+ diagnosis was less than 12 months before study baseline (Yes)	256 (67)	111 (45)	<0.001	139 (57)	92 (43)	0.004	117 (83)	19 (54)	<0.001
Reason for test at time of first HIV-positive test result									
Self-initiated: sickness related	237 (62)	91 (37)	<0.001	137 (56)	74 (35)	<0.001	100 (71)	17 (49)	0.02
Self-initiated: non-sickness related	81 (21)	79 (32)		50 (20)	63 (30)		31 (22)	16 (46)	
Not self-initiated	67 (17)	77 (31)		57 (23)	75 (35)		10 (7)	2 (6)	
Number of CD4 tests prior to study recruitment:^d^ median (IQR)	0 (0, 1)	1 (0,2)	<0.001	0 (0, 1)	1 (0, 2)	0.002	0 (0, 1)	0 (0, 2)	0.02
Number of months engaged in HIV care prior to recruitment CD4 test: median (IQR)	1 (1, 9)	9 (1,21)	<0.001	2 (1, 11)	10 (1, 22)	<0.001	1 (1, 5)	2 (1, 12)	0.15
CD4 at baseline: median (IQR) (4 missing)	133 (76, 175)	632 (559, 768)	<0.001	145 (85, 182)	619 (554, 775)	<0.001	121 (63, 161)	666 (581, 747)	<0.001
Has knowing that you are HIV-positive changed your interest in sex (Yes)	203 (53)	94 (38)	<0.001	120 (50)	83 (39)	0.02	83 (59)	11 (32)	0.005

a,b,c Chi-square test of ART-eligible vs. pre-ART; ^a^overall, ^b^among females, ^c^among males.

d Excluding the recruitment CD4 test.

The majority (69%) of each group answered positively the question “Since you found out you were HIV positive, have you changed your sexual behaviour in any way?”, in the following ways: “used more condoms”, “reduced number of partners”, “stopped having sex”, “talked to partner about HIV”, “been faithful to one partner” or “other.” Comparing the pre-ART to the ART-eligible group, a larger proportion reported using more condoms (71 vs. 58%, *p*<0.001), and talking to their partner about HIV (31 vs. 14%, *p*<0.001), but fewer had abstained from sex (21 vs. 40%, *p*<0.001) or changed their interest in sex (38 vs. 53%, *p*<0.001). The change in interest in sex due to HIV most frequently reported by both groups was “a decreased interest” and “feeling unwell” with both reported more frequently by the ART-eligible group.

Controlling for gender revealed no difference in the number of lifetime partners or sexual partners in the last six months reported by ART group, but the pre-ART group was significantly more likely to have been sexually active in the last month than the ART-eligible group (Table 3). There was no significant difference in reported coital frequency in the last month between ART groups, except among men where the median number of sex acts in the pre-ART group was twice that of the ART-eligible group (4 vs. 2, *p*=0.005). In each group, 73% reported consistent condom use in the last month (*p*=0.31). Table 4 shows the final multivariable model for being sexually active in the last month among those reporting at least one on-going partnership at study baseline. The pre-ART group remained significantly more likely to be sexually active in the last month than the ART-eligible group, aOR: 2.06 95% CI (1.32, 3.21).

**Table 3:** Sexual behaviour and partnership characteristics and perceived self-efficacy by ART group

	Overall			Females			Males		
						
	ART-eligible *N*=385	Pre-ART *N*=247		ART-eligible *N*=244	Pre-ART *N*=212		ART-eligible *N*=141	Pre-ART *N*=35	
						
	*n* (% of *N*)	*n* (% of *N*)	*p* a	*n* (% of *N*)	*n* (% of *N*)	*p* b	*n* (% of *N*)	*n* (% of *N*)	*p* c
Lifetime partners			<0.001			0.27			0.71
Median (IQR)	3 (2, 6)	3 (2, 4)		3 (2, 4)	3 (2, 3)		9 (5, 13)	10 (5, 15)	
Missing *n* (% of *N*)	14 (4)	1 (0)		2 (1)	0 (-)		12 (9)	1 (3)	
Number sexual partners in last 6 months									
0	128 (33)	59 (24)	0.008	83 (34)	53 (25)	0.11	45 (32)	6 (17)	0.17
1	245 (64)	185 (75)		160 (66)	158 (75)		85 (60)	27 (77)	
2 +	12 (3)	3(1)		1 (0)	1 (0)		11 (8)	2 (6)	
Number sexually active in last month before baseline^d^	148 (58)	141 (75)	<0.001	92 (57)	118 (74)	0.002	57 (59)	23 (79)	0.05
Number of sex acts in last month before baseline^d^,^e^: median (IQR)	2 (1, 3)	2 (1, 4)	0.14	2 (1, 3)	2 (1, 3)	0.35	2 (2, 3)	4 (3, 6)	0.005
A condom was used during every sex act in last month before baseline (Yes)^d^,^e^	108 (73)	102 (73)	0.31	67 (74)	84 (72)	0.35	41 (71)	18 (78)	0.89
Disclosed HIV status to their partner (Yes)^f^,^g^	208 (72)	139 (69)	0.46						
Know partner's status (Yes)^e^,^f^	120 (42)	98 (49)	0.12	54 (32)	78 (46)	0.008	66 (55)	20 (63)	0.40
HIV concordancy within relationship^f^			0.29			0.03			0.51
Concordant HIV +	104 (36)	85 (42)		47 (28)	69 (41)		57 (48)	16 (52)	
Discordant	16 (6)	13 (7)		7 (4)	9 (5)		9 (8)	4 (13)	
Partner status unknown	167 (58)	102 (51)		114 (68)	91 (54)		53 (44)	11 (35)	
Used a condom at last sex (Yes)^f^,^g^	173 (60)	124 (62)	0.72						
Condom use at last sex by HIV concordancy^f^, *N* (% using condom):			0.71^h^						
Concordant HIV +	104 (75)	85 (75)							
Discordant	16 (56)	13 (77)							
Partner status unknown	167 (52)	102 (49)							
Reported having used physical violence at least once towards current partner^f^	30 (10)	20 (10)	0.87						
Reported current partner used physical violence at least once in relationship^f^	28 (10)	28 (14)	0.15						
Recently argued (Yes)^f^	97 (34)	56 (28)	0.18						
*Self-efficacy*									
I am certain that I could discuss my HIV-positive status with a sexual partner (agree)	367 (95)	236 (95)	0.94						
I am confident suggesting using condoms with sexual partners (agree)	371 (96)	235 (95)	0.41						
I am confident that I could refuse unsafe sex with a sexual partner even if they were pressuring me to be unsafe (agree)	370 (96)	231 (94)	0.05	234 (96)	197 (93)	0.24	136 (96)	34 (97)	0.58
I am certain that I will have sex with a condom when a sexual partner becomes a regular partner (agree)	370 (96)	215 (87)	<0.001	232 (95)	183 (86)	0.005	138 (98)	32 (91)	0.07
Partner status wanted (3 missing)			<0.001			<0.001			0.43
HIV-positive, on ART	113 (30)	28 (11)		74 (31)	23 (11)		39 (28)	5 (14)	
HIV-positive, not on ART	24 (6)	22 (9)		13 (5)	19 (9)		11 (8)	3 (9)	
HIV-negative	61 (16)	23 (9)		43 (18)	17 (8)		18 (13)	6 (17)	
Their actual HIV status would not matter but I would want to know it	176 (46)	168 (68)		107 (44)	147 (69)		69 (49)	21 (60)	
Not important at all	8 (2)	6 (2)		5 (2)	6 (3)		3 (2)	0 (-)	

a,b,c Chi-square test of ART-eligible vs. pre-ART; ^a^overall, ^b^among females, ^c^among males.

d Of those who reported at least one sexual partner in the last 6 months.

e Overall, no ART group difference, but the gender-specific data are provided because this variable was significantly different between groups within gender.

f Among the 487 relationships on-going at baseline.

g There was no significant difference by ART group in a logistic regression model adjusting for age and gender.

h Test for homogeneity.

**Table 4:** Final multivariable logistic regression model for being sexually active in the last month among those who reported at least one current partner at study baseline (*N*=468)

Variable	*N* (% sexually active in last month)	Unadj OR, 95% CI	Adj OR, 95% CI^a^	*p* b
Study group				
ART	271 (55)	1.0	1.0	0.001
MON	197 (71)	2.04 (1.38, 3.01)	2.06 (1.32, 3.21)	
Currently employed				
No	352 (58)	1.0	1.0	0.004
Yes	116 (73)	2.01 (1.27, 3.20)	2.11 (1.26, 3.52)	
Reason for test at time of first HIV-positive test result				
Self-initiated – sickness related	220 (53)	1.0	1.0	0.0002
Self-initiated – non-sickness related	129 (78)	3.01 (1.80, 5.06)	3.19 (1.86, 5.47)	
Not self-initiated	119 (61)	1.32 (0.83, 2.12)	1.30 (0.77, 2.21)	
Disclosed HIV status to their partner				
No	129 (45)	1.0	1.0	<0.0001
Yes	339 (68)	2.58 (1.71, 3.91)	2.37 (1.49, 3.78)	
Partner resides				
With member	227 (71)	1.0	1.0	0.005
In isigodi	43 (63)	0.68 (0.34, 1.33)	0.72 (0.34, 1.53)	
Outside isigodi	198 (50)	0.40 (0.27, 0.60)	0.42 (0.26, 0.67)	
Discussed having another child with partner				
No	300 (58)	1.0	1.0	
Yes	168 (68)	1.53 (1.03, 2.27)	0.73 (1.10, 2.71)	0.02

a Final model also adjusted for gender (Wald test *p*=0.57) and age as a categorical variable (Wald test *p*=0.30).

b Likelihood ratio test p-value.

In the two groups combined, 487 partnerships were on-going at study baseline involving 468 participants, with 36 (7%) of the relationships reported as not sexually active at baseline. In 71% of the partnerships, participants reported to have disclosed their HIV-positive status to their partner before recruitment to the study, and 61% reported using a condom at last sex (with no difference between groups in models with age and gender, *p*=0.96 and *p*=0.48, respectively). Among the pre-ART group, the participant knew their partner's HIV status in 49% of partnerships, compared to 42% among the ART-eligible group (*p*=0.12); however, in a multivariable logistic regression model (Table 5), the pre-ART group was twice as likely to know their partners’ HIV status than the ART-eligible group, aOR 1.95 (1.18, 3.22) after additional adjustment for whether the participant had disclosed their own HIV status to their partner, perceived their partner to have had sex with other people in the last six months, cohabited with their partner, discussed having another child with their partner, used a condom at last sex, and gender norms score. An interaction term between gender and group was not significant, *p*=0.68. None of the other partner characteristics or relationship-level variables were significant in the final model. Overall, 39% of the study participants knew they were in concordant HIV-positive relationships. Condom use at last sex was significantly lower in both ART groups among those who did not know their partners’ HIV status.

**Table 5:** Final multivariate logistic regression model for knowing partner's HIV status among 487 relationships that were on-going at baseline

Variable	N (% knowing partner's status)	Unadj OR, 95% CI	Adj OR, 95% CI^a^	*p* b
Study group				
ART	287 (42)	1.0	1.0	0.009
MON	200 (49)	1.34 (0.92, 1.94)	1.95 (1.18, 3.22)	
Disclosed HIV status to their partner				
No	141 (9)	1.0	1.0	<0.0001
Yes	346 (59)	14.32 (7.79, 26.31)	13.42 (7.19, 26.93)	
Partner resides				
With member	226 (61)	1.0	1.0	0.005
In isigodi	49 (24)	0.21 (0.10, 0.44)	0.30 (0.13, 0.69)	
Outside isigodi	212 (33)	0.31 (0.21, 0.47)	0.51 (0.30, 0.87)	
Gender norm quartiles:				0.01
Least support for equitable norms (31–37) 1	137 (45)	1.0	1.0	
(38–41) 2	154 (51)	1.24 (0.78, 1.99)	1.52 (0.84, 2.77)	
(42–45) 3	84 (29)	0.48 (0.27, 0.88)	0.47 (0.22, 1.01)	
Most support for equitable norms (46–57) 4	112 (48)	1.13 (0.67, 1.88)	1.42 (0.72, 2.77)	
Has partner had sex with others in last 6 months				
No	175 (59)	1.0	1.0	0.0006
Do not know	157 (47)	0.59 (0.38, 0.92)	0.88 (0.51, 1.52)	
Yes, I think so/yes, definitely	155 (26)	0.25 (0.15, 0.39)	0.34 (0.19, 0.61)	
Condom use at last sex				
No	190 (30)	1.0	1.0	0.004
Yes	297 (54)	2.76 (1.86, 4.10)	2.04 (1.25, 3.33)	
Discuss having another child with partner				
No	314 (43)	1.0	1.0	0.05
Yes	173 (49)	1.27 (0.87, 1.85)	1.62 (1.0, 2.65)	

a Final model also adjusted for gender (*p*=0.10) and age as a categorical variable (*p*=0.44).

b Adjusted Wald test *p*-value.

c Variance adjusted for cluster correlation among participants in multiple on-going relationships.

Almost all respondents in both ART groups reported that they would be able to discuss their HIV-positive status, refuse unsafe sex with a sexual partner even if they were being pressured to be unsafe, and suggest using condoms with a new sexual partner, yet approximately 30% in both groups had not disclosed in their current relationship. Controlling for gender, the pre-ART group was significantly less certain that they would have sex with a condom when a sexual partner becomes a regular partner compared to the ART-eligible group. When asked whether knowing the HIV status of a future partner would be important to them, 97% said it would be very important (no differences by group, *p*=0.60). However, the specific desired HIV status of a future partner differed between the two groups, with 68% of the pre-ART group and 46% of the ART-eligible group saying the partner's status would not matter as long as they knew it (*p*<0.001). More of the ART-eligible group wished a future partner to be HIV-positive and on ART (Table 3). However, among individuals in on-going relationships who said it would be very important to them to know a *future* partner's HIV status, only 55% knew their *current* partner's status and this was not statistically different between ART groups (*p*=0.27)’.

## Discussion

This study demonstrates that pre-ART individuals with CD4>500 cells/µl and ART-eligible individuals have similar socio-demographic characteristics, except for current sexual behaviour and HIV-testing history. Participants in the pre-ART group are twice as likely to be sexually active in the last month and twice as likely to know their partner's status, compared to ART-eligible individuals. Despite the pre-ART group reporting more frequently that they had increased condom use and talking to their partner about HIV since learning their HIV status, condom use and HIV disclosure to their partner were reported at the same levels as the ART group. Overall, 71% reported disclosure of their HIV status to their current sexual partner and 61% to using a condom at last sex. These risk behaviours occur in spite of risk-counselling that occurs at the clinics. Barriers to safer sex for people living with HIV include fear of disclosure, negative attitudes to condoms, lack of partner support and fertility intentions [25]. The proportion of participants not knowing their current partner's HIV status (55%) is higher than previously reported among a HIV-positive cohort in Soweto, South Africa [26]. As in the Venkatesh *et al*. study, women in our study are significantly less likely to know their partner's status than men. In our study, only half of those not knowing their partner's status report using a condom at last sex. These findings suggest that facilitating partner testing and alternative HIV prevention options for safer sex would be beneficial. The risk behaviours of the CD4>500 cells/µl group are of particular concern because current WHO guidelines only provide ART to HIV-infected people with CD4<350 cells/µl [11,12]. Thus, people with CD4>500 cells/µl will remain a key group contributing to the continued spread of HIV. The proposed treatment-as-prevention strategy whereby HIV-infected individuals are initiated on ART as soon as they are identified irrespective of CD4 count, may reduce the risk of onward transmission in this group [27].

In contrast to the previous studies focusing on the impact of ART use on sexual risk behaviours in Africa [2–4], our study compares sexual behaviours of ART-naïve individuals accessing HIV care at earlier vs. later stages of HIV disease according to their CD4 counts. In multivariate analysis, the pre-ART group are twice as likely to be sexually active in the last month indicating that illness (represented by lower CD4 count) is associated with less sexual activity. Venkatesh *et al*. used data from an urban HIV clinic in Soweto, South Africa and considered ART status as a time-varying covariate, comparing the pre-ART period to the on-ART period [1]. The authors found that the pre-ART period was associated with significantly increased odds of being sexually active compared to the on-ART period. Assuming the pre-ART period in their study represents, on average, higher CD4 counts than the on-ART period, their results are consistent with our study findings.

In our study groups, disclosure levels within current partnerships did not match the perceived self-efficacy of individuals to disclose to future partners. Whether this reflects issues related to the current relationship that the individual believes will not affect future relationships, or whether participants are unrealistic about future relationships, is not possible to discern in this study. Among the numerous relationship attributes and partner characteristics collected, few were significantly associated with HIV disclosure and knowledge of partner status. Cohabitation with a partner, having discussed whether to have another child with that partner, and being confident that a partner had not recently had sex with someone else, were all associated with knowledge of partner status. This suggests that couple-focused interventions that improve communication and the quality of relationships may facilitate mutual HIV disclosure within couples and reductions in sexual risk behaviours [28].

The pre-ART group sought health care earlier than the ART-eligible group as measured by participants’ HIV-testing history. This was beneficial for the individual's own health and crucial for the broader community, both for opportunities to reduce onward transmission but also to normalize testing and care in the community. In contrast, many of the ART-eligible group presented late to the HIV clinic, with a sickness-related reason for testing, suggesting that there is a need to further promote HIV testing in the community to assist earlier diagnosis. A recent South African HIV counselling and testing campaign tested over 13 million South African adults by June 2011, and the South African National Strategic Plan for 2012–2016 calls for universal HIV testing of every South African 12 years and older (sexually active, with previous HIV-negative test, or of unknown status) on an annual basis [29,30]. Knowledge of status may motivate greater responsibility among HIV-positive individuals towards preventing transmission to sexual partners [31].

There are a number of limitations to this study. No information was available about individuals who presented for a CD4 test but did not return for their results. It is possible that the pre-ART individuals in this study do not represent all individuals with CD4>500 cells/µl who access the programme at least once. However, the gender and age balance of this study group is consistent with the demographic profile of the larger programme [6]. The ART-eligible group is also broadly representative of ART-initiators in the local programme [23] and in eight public-sector programmes in South Africa [32]. It is possible that social desirability influenced the self-reports of sexual behaviours in this study. Other studies in Africa report that men tend to over- and women under-report sexual partnerships [33–35]. However, a sizable proportion of respondents in this study still report sexual behaviours known to be a risk for onward HIV transmission, despite standard counselling and prevention messaging at the clinic, suggesting that social desirability is relatively low in this rural population. Information about partners was provided by the participant and may have been inaccurate and contributed to the lack of association in this study between most relationship and partner characteristics and the outcomes of interest. The cross-sectional nature of these analyses prevents the examination of causality.

A priori, we had hypothesized that individuals with CD4>500 cells//µl accessing HIV care would differ from ART-eligible individuals in terms of psychosocial characteristics. However, the majority of the social support and social capital variables measured, and the stigma and gender norms scores, did not differ significantly between the two ART groups. The pre-ART group appears to have no more access to social support and social capital than the ART-eligible group and be equally in need of support from the community prior to accessing ART. The types of support that communities provide to people initiated on ART include facilitating adherence, physical care and recognizing side effects [36–38]. Effective ways of community support for pre-ART individuals are less clear, but could include supporting pre-ART individuals to return for regular CD4 testing and providing counselling support to facilitate partner testing. This is an area that would benefit from further research.

Our study indicates a continued risk of onward HIV transmission by pre-ART individuals with CD4>500 cells/µl and highlights the need for positive prevention efforts to target this group. Our results also demonstrate a need to strengthen messaging and interventions regarding disclosure, partner HIV testing and consistent condom use among all individuals who access the HIV treatment and care programme.
